# Imaging neuroendocrine tumours of the pancreas: role of CT and MRI

**DOI:** 10.1186/1470-7330-14-S1-O28

**Published:** 2014-10-09

**Authors:** Anju Sahdev

**Affiliations:** 1 Department of Imaging, St Bartholomew’s Hospital, Barts Health, London EC1A 7BE, UK

## 

Pancreatic neuroendocrine tumours (pNETS) are a part of a heterogeneous group of tumors, neuroendocrine gastroenteropancreatic tumors (GEP-NETs), with their origin in neuroendocrine cells of the embryological gut. Most commonly, primary lesions are located in gastric mucosa, small and large intestine, rectum and pancreas. The overall incidence of all GEP-NETS has increased over the past decade, with pNET incidence of 0.32/100 000/year. Patients with multiple endocrine neoplasia type 1 (MEN-1) or von Hippel–Lindau’s disease (VHL), have pNETS 15–20 years earlier than patients with sporadic pNETS [1].

CT and MRI are typically the first line imaging modalities of choice in the evaluation of most patients with suspected pNETS. The role of cross sectional imaging is complementary to somatostatin receptor based imaging and PET CT. CT and MRI are essential in the detection of primary tumours, which is challenging in small secretory insulinomas and gastrinomas. Meticulous care must be taken in cross sectional imaging technique to maximize the sensitivity for detection of the small and frequently multiple lesions. CT imaging should include triphasic imaging. No one sequence detects all pNETS on MRI and multiple sequences including T1, T2, fat saturated images are required. Over the last few years the use of diffusion weighted imaging (DWI) has also improved primary lesion detection in the pancreas [2] and detection of small metastatic liver and bone lesions.

Surgery is the only curative treatment for pNETS . The role of CT and MRI also extends to staging and planning surgical resection. Excellent anatomical detail can be accurately provided with CT and multi-planar and thin section imaging. The staging for pNETS is based on recommendations from European Neuroendocrine Tumour Society [3] and WHO staging which will be outlined with imaging and pathological illustrations.

Larger pNETS, usually have low or no secretory activity and characteristic features on CT and MRI, which provide an indication to their histopathology. Punctate calcifications, lack of biliary obstruction and vascular invasion despite large tumour volume are features that allow the radiologist to make the initial working diagnosis of a pNET. In these patients the role of imaging is to provide tissue diagnosis and staging by biopsy, assess tumour bulk, detect distant metastases and assess disease response following therapy.

A large number, close to 50%, of non-secretory pNETS present with distant disease. These are offered radiolabelled therapies but increasingly radiological embolization techniques and radiofrequency is available for local control. Targeted therapies with vascular endothelial growth factor receptor-tyrosine kinase inhibitor (VEGFr-TKI), Sunitanib and the mammalian target of rapamycin (mTOR) inhibitor, Everolimus for medical management of locally advanced and metastatic disease sees the role of CT and MRI now extending to evaluation of disease response in phase II and III trials. The imaging techniques again require scan phase precision and consistency to ensure accuracy for tumour response assessment which is based on RECIST criteria.
